# Typhoid Conjugate Vaccines and Enteric Fever Control: Where to Next?

**DOI:** 10.1093/cid/ciaa343

**Published:** 2020-07-29

**Authors:** A Duncan Steele, Megan E Carey, Supriya Kumar, Calman A MacLennan, Lyou-Fu Ma, Zoey Diaz, Anita K M Zaidi

**Affiliations:** Enteric and Diarrheal Diseases, Bill & Melinda Gates Foundation, Seattle, Washington, USA

**Keywords:** enteric fever, typhoid, typhoid conjugate vaccines, disease control

## Abstract

After the unprecedented success and acceleration of the global agenda towards typhoid fever control with a strong World Health Organization recommendation and the approval of funding from Gavi, the Vaccine Alliance (Gavi), for the use of a new typhoid conjugate vaccine (TCV), we should turn our minds to the challenges that remain ahead. Despite the evidence showing the safety and clinical efficacy of TCV in endemic populations in developing countries, we should remain vigilant and explore hurdles for the full public health impact of TCV, including vaccine supply for the potential global demand, immunization strategies to optimize the effectiveness and long-term protection provided by the vaccines, potential use of TCV in outbreak settings, and scenarios for addressing chronic carriers. Finally, challenges face endemic countries with poor surveillance systems concerning awareness of the need for TCV and the extent of the issue across their populations, and how to target immunization strategies appropriately.

## ENTERIC DISEASE BURDEN ESTIMATES ARE IMPROVING

Disease burden estimates are crucial to our long-term understanding of enteric fever and appropriate intervention strategies. Without global, regional, and national estimates of the burden of enteric fever, questions persist about where and how to use vaccines, how much vaccine is required for manufacturers to produce, what financial resources are required to invest in the procurement of vaccine for Gavi, the Vaccine Alliance (Gavi)–eligible and vulnerable communities, and how the global health community should strategize a cohesive and feasible approach to typhoid fever control. Our estimates are getting better.

New model-based estimates of the burden of enteric fever caused by *Salmonella enterica* subspecies enterica serovar Typhi (*S*. Typhi) suggest approximately 10.9 million typhoid cases (95% confidence interval [CI], 9.3–12.5 million) occurred in 2017, resulting in 117 000 deaths and 8.437 million (4.731–13.577 million) disability-adjusted life-years (DALYs) [1]. The highest age-specific incidence rate of the disease is in children between 5 and 9 years of age [1], and 12.6% (95% CI, 8.7–17.7%) of cases occur in children younger than 5 years of age, highlighting the importance of early immunization strategies. The Global Burden of Disease (GBD) enteric fever global incidence estimates are expected to become more robust with the continued incorporation of data from ongoing large, regional surveillance studies in sub-Saharan Africa and South Asia.

Furthermore, these regional surveillance networks will give us better national and subregional data for country awareness and consideration. The Severe Typhoid Fever in Africa (SETA) Study, following on from the Typhoid Fever Surveillance in Africa Program (TSAP) [2], is generating incidence estimates for severe typhoid fever in sub-Saharan countries that did not have data before, and demonstrating that parts of East and West Africa have a high burden of typhoid that is comparable to that in South Asia. Combined data from TSAP and the SETA study give almost a decade of data from some countries and are being used to inform Ministries of Health.

Similar ongoing studies in South Asia include surveillance in Pakistan, Nepal, and Bangladesh (Surveillance for Enteric Fever Asia Project [SEAP]), and India (Surveillance for Enteric Fever in India [SEFI]). These studies corroborate the high disease incidence in all countries studied and additionally differentiate between *S*. Typhi and *Salmonella enterica* subspecies *enterica* serovar Paratyphi (*S.* Paratyphi) A, B, or C as the cause of enteric fever. Enteric fever is caused by *S.* Typhi and serovars *S.* Paratyphi A, B, and C. These Paratyphi serovars, of which *S.* Paratyphi A is the most commonly isolated, were estimated using etiological proportion models, to cause 3.40 (2.666–4.184) million cases, 1.364 (0.631–2.641) million DALYs, and 19 000 (8 700–37 000) deaths in 2017 [2]. Incorporation of data from the SEAP and SEFI studies will enable the GBD to provide incidence, DALY, and mortality estimates for typhoid and paratyphoid separately. This is important information as we consider whether a bivalent vaccine approach is appropriate for *S.* Typhi and *S.* Paratyphi A.

## COMPLICATIONS OF CLINICAL ENTERIC FEVER DISEASE

In a small proportion of those infected with *S.* Typhi, infection can result in chronic carriage of the bacterium in the gall bladder, and prolonged intermittent shedding. Chronic typhoid carriage is associated with gall bladder cancer later in life [3]. Typhoid DALY estimates should incorporate this association between *S.* Typhi infection and gall bladder cancer, which is currently underresearched and difficult to identify. The association is being further investigated, incorporating global data including a rich dataset from Santiago, Chile [3], and the outputs will be incorporated into future GBD estimates of typhoid DALYs.

Severe typhoid disease can also result in intestinal perforations [4, 5], a life-threatening condition in populations with inadequate access to appropriate surgical interventions. This association is being further explored in the regional surveillance networks, SEAP, SEFI, and SETA, and will increase our understanding of the association with *S.* Typhi infection. These new data should help refine global estimates of enteric fever burden, and importantly, should inform national decisions regarding typhoid conjugate vaccine (TCV) introduction.

## WILL THE IMPROVING DATA BE UTILIZED BY COUNTRIES?

Whereas typhoid and paratyphoid burden data are becoming available from more countries than ever before, these are generated in large part by externally funded multicenter surveillance studies. It is unclear if countries will assume the costs of ongoing surveillance after these studies end. Moreover, many countries do not have the capacity to conduct blood culture isolation or surveillance for *S.* Typhi. The rapid diagnostic tests currently available are known to have poor positive predictability. Blood culture is the accepted standard for appropriate detection of the pathogen despite its 50–60% positive predictive reliability [6]. However, this is not only costly and requires skilled, trained technicians but also requires standardized criteria for selecting and testing patients presenting with a fever.

A low-cost tool to help estimate the population burden of typhoid and paratyphoid could benefit low- and middle-income countries (LMICs) greatly. Serological markers have shown some promise [7, 8], and efforts continue to validate multiple markers to estimate *S.* Typhi burden. A reliable, robust tool is urgently required for diagnosis and to provide country Ministries of Health with national data for decision making and for targeted interventions to communities at high risk.

Efforts are also underway to develop a tool to conduct environmental surveillance for *S.* Typhi, which, as a human-restricted pathogen, has shown the propensity to survive in the environment between human infections, and may be detected in sewage and effluent water during this inter-infection period. The bacterium has been shown to enter into a viable but nonculturable state, making it difficult to revive in the laboratory with known culture media [9, 10]. However, molecular methods may be useful to detect and quantify the bacterium if these challenges can be overcome. A standardized protocol to collect and analyze sewage samples could be useful to estimate circulation and persistence of *S.* Typhi in resource-poor, low-income settings. Data from environmental surveillance may also be considered as part of a country’s application for Gavi funding for new vaccine introduction [11].

Such a tool may be especially useful for countries to plan phased introduction of TCV by ensuring early access to vaccine in areas with the highest potential burden. Systematic sewage surveillance has been undertaken as part of poliovirus surveillance alongside clinical surveillance for acute flaccid paralysis, and is being expanded as part of the endgame in polio eradication [12–14]. Although important differences exist between the use cases for environmental surveillance for polio and typhoid, it would be of great benefit if the existing polio environmental surveillance system could be leveraged for other pathogens in future.

## BUILDING THE COUNTRY EVIDENCE BASE FOR TYPHOID CONJUGATE VACCINE IMMUNIZATION

In addition to blood culture surveillance, serological surveillance, and environmental surveillance data, the presence of nontraumatic ileal intestinal perforations (a potential complication of typhoid [15, 16]) in hospitals, could also support a country’s decision to introduce TCV. In communities with inadequate access to surgery, intestinal perforations can be life-threatening, in addition to placing a high financial burden on affected families. A specific case definition of intestinal perforations causally associated with *S.* Typhi infection could be extremely useful in countries attempting to understand the extent of their typhoid burden. Interestingly, high levels of intestinal perforations in Liberia, alongside high-risk factors known to be associated with enteric fever, and surveillance data from neighboring countries, persuaded the government to apply to Gavi for support in TCV introduction. The application was approved on the basis of this set of indirect data.

## ANTIMICROBIAL RESISTANCE IN *S*. TYPHI

Extensively drug-resistant (XDR) *S.* Typhi, which is resistant to chloramphenicol, ampicillin, trimethoprim–sulfamethoxazole, fluoroquinolones, and third-generation cephalosporins, has been spreading in Pakistan [17] and is, unsurprisingly, leading to longer hospital stays and higher health systems costs [18]. Single nucleotide polymorphisms have been shown to be associated with azithromycin resistance in *S*. Typhi and *S*. Paratyphi A in Dhaka, Bangladesh [19]. Whether these strains are comparable in fitness, transmissibility, survival in the environment, and rates of chronic carriage to antibiotic-susceptible strains of *S.* Typhi is unknown, but this information has implications for the predicted impact of TCV on reducing the spread of typhoid and on antimicrobial resistance in general [20, 21].

Chronic carriage of *S.* Typhi in the gall bladder results from the formation of biofilms [22], a process that has recently been shown to be compromised in antibiotic-resistant strains of *Salmonella enterica* subspecies *enterica* serovar Typhimurium [23]. Understanding whether *S.* Typhi strains are variable in their potential for chronic carriage, environmental survival, and transmission, as well as evaluating the impact of TCV introduction on potential serotype replacement, antibiotic use, and the development/persistence of resistant strains, remain important research questions to address.

## TYPHOID CONJUGATE VACCINES ARE READY FOR PUBLIC HEALTH USE

The global health agenda has long awaited a TCV to utilize as a public health tool with the ability to vaccinate infants and younger children and an anticipated longer duration of protection than the Vi polysaccharide and the Ty21a live-attenuated typhoid vaccines. It is well recognized that longer-term investments in safe water, improved sanitation, and behavioral changes at the household level are the ultimate goal for sustainable enteric fever control and prevention. Nevertheless, TCVs are currently available for short-term impact and prevention of the disease and have demonstrated the ability for safe and effective control in specific endemic settings.

Currently, there are 4 Indian licensed, commercially available typhoid conjugate vaccines—Typbar-TCV (Bharat Biotech India Ltd, Hyderabad), PedaTyph (Bio-Med Ltd, India), ZYVAC TCV (Zydus Cadila, India) [24], and the recently licensed Vi-CRM_197_ (Biological E Ltd, India). Currently, only Typbar-TCV is a World Health Organization (WHO) prequalified vaccine. All have been licensed on immunogenicity data utilizing thresholds for clinical protection established by a novel TCV in the 1990s. The original TCV was developed at the US National Institutes of Health consisting of the Vi-polysaccharide capsular protein conjugated to a recombinant exotoxin from *Pseudomonas aeruginosa* (rEPA) vaccine [25]. This Vi-rEPA vaccine was produced by Lanzhou Institute of Biological Products, Lanzhou, China, and evaluated in a large phase 3 efficacy study in Vietnam [26]. Despite the early implementation of this vaccine in China, the commercial production, licensure, and broader public health utility of the product have stalled.

## TYPHOID CONJUGATE VACCINES FOR GLOBAL PUBLIC HEALTH

Thus, from a global public health viewpoint, there is only 1 TCV that is prequalified by WHO and available for procurement by UNICEF and Gavi. Typbar-TCV consists of the Vi polysaccharide conjugated to a tetanus toxoid carrier protein (Vi-TT) and was licensed based on immunogenicity studies conducted in India [27]. The clinical protection afforded by the vaccine was evaluated in an adult challenge study in Oxford, United Kingdom, showing high efficacy against clinically presenting disease [28], which supported both the WHO prequalification of the product and a strong recommendation for its use by the WHO Strategic Advisory Group of Experts (SAGE).

Subsequently, significant additional data have been generated on the safety and immunogenicity of Typbar-TCV from prelicensure studies conducted by the manufacturer [27] and postmarketing surveillance conducted in the private sector in India. These safety data were reviewed by the WHO Global Advisory Committee on Vaccine Safety in December 2018, which deemed the safety profile of Typbar-TCV to be acceptable [29]. Furthermore, safety and efficacy data from the ongoing studies being conducted by the Typhoid Vaccine Acceleration Consortium (TyVAC) in Nepal, Malawi, and Bangladesh will contribute to the public health use of the vaccine [30]. A randomized clinical trial evaluating the efficacy of Typbar-TCV in children between 9 months and 15 years of age in Nepal [31] showed high efficacy for the first time in children in an endemic setting.

## TYPHOID CONJUGATE VACCINES ARE BEING USED IN THE PUBLIC HEALTH SECTOR

Typbar-TCV is currently being implemented in the public sector. In Pakistan, the XDR *S.* Typhi circulating in Hyderabad city in Sindh province led to the vaccination of children to limit the spread of the strain [32]. In neighboring India, the municipal authorities in Navi Mumbai made the decision to introduce the vaccine in children based on observed high rates of enteric fever [33]. Using a step-wedge design for immunization, an estimate of vaccine impact on reducing the burden of disease in vaccinated communities compared with control communities will be determined. There is substantive reason to expect that TCVs will make a significant impact on disease burden when used at scale as well as having the potential to curb the spread of resistant strains; more empirical impact data will be forthcoming over the next year.

## THE TYPHOID CONJUGATE VACCINE PIPELINE IS ROBUST

While there is currently only 1 WHO prequalified TCV available for UNICEF procurement, there is a rich pipeline of additional TCV candidates in clinical development that should contribute to vaccine supply security if they obtain WHO prequalification over the next few years [24]. The accelerated pathway to licensure based on immunogenicity bridging data to the WHO prequalified Typbar-TCV for ultimate prequalification is being utilized by these other TCV manufacturers but will require additional large-scale postmarketing studies.

The International Vaccine Institute, Seoul, Republic of Korea, has developed a Vi polysaccharide vaccine construct conjugated to diphtheria toxoid (Vi-DT) [24], and this technology has since been transferred to SK Bioscience in South Korea, PT BioFarma in Indonesia, and Incepta Ltd in Bangladesh. Both SK Bioscience and PT BioFarma have recently completed phase 1 and 2 studies with robust immune responses [24] and have started enrollment into large phase 3 pivotal studies, for anticipated national licensure within the next 2 to 3 years, and WHO prequalification approximately 12 months later.

An alternative construct with Vi-polysaccharide from *Citrobacter freundii* conjugated to CRM_197_ was developed by the Novartis Vaccines for Global Health (now GSK Vaccines Institute for Global Health) [34], and the technology subsequently transferred to Biological-E in Hyderabad, India. Following manufacture by Biological-E, Vi-CRM_197_ has been evaluated in phase 1 and Phase 2/3 studies in India and is currently undergoing further phase 3 studies. Indian licensure was granted in 2020 and WHO prequalification will be applied for. Thus, we have 3 new TCVs, albeit with different carrier proteins, on the verge of entering the global public health arena.

## UNCERTAINTY AROUND THE GLOBAL DEMAND FOR TYPHOID CONJUGATE VACCINES

Although 4 vaccine manufacturers are actively developing TCVs and planning for entry into the global market, there remains some uncertainty about what the future global demand for TCVs will be. Part of this is driven by lack of clarity about typhoid burden in many areas, as described above, given the lack of robust, inexpensive tools to estimate burden and systematic blood culture surveillance in most countries. In addition, there remain limited data from some regions, such as Latin America and North Africa and the Middle East, and some large countries. Typhoid cases have occurred in these regions demonstrating the presence of risk factors and the circulation of the pathogen; in addition, multidrug-resistant *S.* Typhi strains have been recorded [35].

Further uncertainty comes from choice of vaccination strategy by countries—WHO SAGE has recommended inclusion of TCV in the National Immunization Program at 9 months of age with measles vaccine, and one-off catch-up campaigns in children up to 15 years of age in endemic regions [36]. Countries must decide which age groups and subnational regions or at-risk communities to target with vaccine doses. In addition, typhoid fever can cause focal outbreaks, which further contributes to uncertainty around required vaccine supply.

TyVAC is supporting decision makers in countries to assess their local need for TCVs and to decide on the context-optimal immunization strategy to implement. These frequent interactions with stakeholders in Gavi-eligible countries, Gavi, and the vaccine manufacturers help mitigate some demand uncertainty. What is clear, thus far, is that, currently, there is significant country interest in TCV introduction—Pakistan has already started their introduction and Liberia and Zimbabwe will introduce TCV in 2020 with Gavi support. There is considerable interest from other countries, and it is anticipated that several countries will apply to Gavi in 2020 for introduction in 2021 and beyond.

## IMMUNIZATION STRATEGIES FOR TYPHOID CONJUGATE VACCINE

Following a careful review of recent disease incidence data, as well as clinical and health economic data for Typbar-TCV, WHO SAGE recommended introduction of TCV in endemic countries as a single dose in routine immunization, either at 9 months or in the second year of life, accompanied by catch-up campaigns, where feasible, in children up to 15 years of age. The WHO also emphasized the need for TCV use in settings with high levels of antimicrobial resistance [37].

In selecting an optimal vaccination strategy, decision makers in countries should consider not only epidemiological data but also the operational feasibility, sustainability, and cost-effectiveness of a TCV introduction. Following a successful application for support, Gavi is willing to cover the full cost of the catch-up campaign implementation in children aged 9 months to 15 years of age (vaccine doses and operational costs) and co-financing for vaccine doses used in routine immunization. In addition, support is available for the operational costs of integration of the vaccine into existing immunization programs. Ambitious TCV vaccination strategies that include catch-up campaigns among older individuals were found to be the most impactful and cost-effective strategy in areas with moderate to high incidence [38].

Key questions remain about optimal use of TCVs in outbreak-response settings, including the timing of vaccination (how soon after an outbreak is detected should vaccination be conducted in order to be optimally effective) and the ages to be vaccinated (should working adults be included in vaccination strategies given uncertainty about the source of the outbreak and mode of transmission). Lessons can be gleaned from the XDR outbreak immunization campaign in Hyderabad, Pakistan, and from use of TCV in Zimbabwe to curtail an ongoing problem there. These data should inform WHO and Gavi strategies for how to use the vaccine in these situations.

## IS A BIVALENT *S*. TYPHI AND *S*. PARATYPHOID VACCINE NECESSARY?

*S*. Paratyphi A enteric fever is clinically indistinguishable from enteric fever caused by *S*. Typhi but is clearly present in Asia. In Nepal, for instance, the proportion of enteric fever due to *S*. Paratyphi A increased from 25% in the 1990s to 41% in 2013 [39], thus providing a clear rationale for the development of bivalent enteric fever vaccines that can protect against both *Salmonella* serovars. Several bivalent vaccine constructs are in development.

Biological-E currently has a vaccine in development consisting of Vi- CRM_197_ and the *S*. Paratyphi A O:2 antigen, also conjugated to CRM_197_. As with the monovalent Vi-CRM_197_ vaccine, the bivalent was originally technology transferred from the Novartis Vaccines Institute for Global Health [40]. Other vaccine developers with bivalent enteric fever vaccines in development include Bharat Biotech in collaboration with the University of Maryland [41] and the International Vaccine Institute and various Chinese manufacturers including the Lanzhou Institute of Biological Products.

The regulatory pathway for vaccines against *S*. Paratyphi A and the bivalent enteric fever vaccine raises some interesting questions. While field efficacy studies are normally expected for a first vaccine against a specific pathogen such as *S*. Paratyphi A, the incidence of paratyphoid fever makes such studies particularly large. The obvious tethering in bivalent formulation to a TCV that has already been licensed or can be licensed through a noninferiority study to a licensed TCV presents an attractive opportunity for accelerated licensure. In addition, the availability of an *S*. Paratyphi A controlled human infection model at Oxford University [42] allows the possibility for assessment of efficacy of the *S*. Paratyphi A component without resorting to field efficacy studies. Such accelerated regulatory pathways are currently under discussion and may necessitate phase 4 postlicensure studies to confirm vaccine effectiveness.

## WATER, SANITATION AND HYGIENE (WASH) - INTEGRATED STRATEGIES

Enteric fever risk is intimately associated with access to clean water. The Sustainable Development Goal 6 targets the universal and equitable access to safe and affordable drinking water for all with implications for typhoid control [43]. However, in rapidly growing urban areas in many LMICs, the planning, resources, and infrastructure required to achieve this goal are lacking. For example, the urban population in Africa grew from 32 million in 1950 to 491 million in 2015, and from 78 million in 1950 in South Asia to 628 million in 2015 [44]. Climate change and the drying up of water tables in Cape Town, South Africa, and Chennai, India, among other cities [45], have highlighted the need for innovation to guarantee safe water supply in urban developing contexts where rapid urbanization is occurring.

*S.* Typhi has been detected in drinking water sources in Hyderabad, Pakistan; Dhaka, Bangladesh; and Kathmandu, Nepal [46–48] and will require substantial investment and long-term commitment to address. The introduction of TCV is a cost-effective and rapid way to prevent typhoid infections and save lives in the near future.

## SUMMARY

It is an exciting time for typhoid control as the global community enters the era of TCV deployment at scale. While there is an evident rising tide of momentum in addressing the global burden of enteric fever, there is still much work to be done to maximize and sustain the potential health impact. We eagerly await the additional impact data from TCV use at scale in public health programs, to continue to refine the tools available to ascertain the burden of disease and to see additional preventative interventions become available for use in future. Underlying all of the current and future success is a strong network of global and national partners, policy makers, and healthcare workers that make ambitious typhoid control goals a reality.

## Notes

***Financial support*:** No funding was received to perform this work.

***Supplement sponsorship.*** This supplement is funded with support from the Coalition against Typhoid Secretariat, housed at the Sabin Vaccine Institute in Washington, DC and made possible by a grant from the Bill & Melinda Gates Foundation.

***Potential conflicts of interest.*** C. A. M. is a named inventor on a patent (US20160263213A1) for *Salmonella* conjugate vaccines, which is assigned to GSK; C. A. M. has no financial interest in the patent. All other authors report no potential conflicts. All authors have submitted the ICMJE Form for Disclosure of Potential Conflicts of Interest. Conflicts that the editors consider relevant to the content of the manuscript have been disclosed.
